# Dying piece by piece: carbohydrate dynamics in aspen (*Populus tremuloides*) seedlings under severe carbon stress

**DOI:** 10.1093/jxb/erx342

**Published:** 2017-09-27

**Authors:** Erin Wiley, Günter Hoch, Simon M Landhäusser

**Affiliations:** 1 Department of Renewable Resources, University of Alberta, Edmonton, Canada; 2 Department of Environmental Sciences - Botany, University of Basel, Basel, Switzerland

**Keywords:** Tree mortality, carbon limitation, non-structural carbohydrates, starvation, shade, storage, remobilization

## Abstract

Carbon starvation as a mechanism of tree mortality is poorly understood. We exposed seedlings of aspen (*Populus tremuloides*) to complete darkness at 20 or 28 °C to identify minimum non-structural carbohydrate (NSC) concentrations at which trees die and to see if these levels vary between organs or with environmental conditions. We also first grew seedlings under different shade levels to determine if size affects survival time under darkness due to changes in initial NSC concentration and pool size and/or respiration rates. Darkness treatments caused a gradual dieback of tissues. Even after half the stem had died, substantial starch reserves were still present in the roots (1.3–3% dry weight), indicating limitations to carbohydrate remobilization and/or transport during starvation in the absence of water stress. Survival time decreased with increased temperature and with increasing initial shade level, which was associated with smaller biomass, higher respiration rates, and initially smaller NSC pool size. Dead tissues generally contained no starch, but sugar concentrations were substantially above zero and differed between organs (~2% in stems up to ~7.5% in leaves) and, at times, between temperature treatments and initial, pre-darkness shade treatments. Minimum root NSC concentrations were difficult to determine because dead roots quickly began to decompose, but we identify 5–6% sugar as a potential threshold for living roots. This variability may complicate efforts to identify critical NSC thresholds below which trees starve.

## Teaser

Carbon starvation causes gradual tissue die-back in seedlings of *Populus tremuloides*, beginning long before carbohydrate reserves are depleted. When tissues die, starch is depleted, but substantial levels of sugars – which vary between organs – can still remain.

## Introduction

Mechanisms of tree mortality remain poorly understood, hindering our ability to predict forest die-off events (Allen *et al.*, 2010) and to understand how different stressors affect trees and why some individuals or species are able to cope and survive and others are not. Carbon starvation – the process by which a tree dies as the result of insufficient carbon to support the metabolism, defense, and other basic maintenance and repairs necessary for sustaining life – is potentially involved in tree death resulting from many factors including drought, defoliation, and decline-diseases (Bamber and Humphreys, 1965; McDowell *et al.*, 2008, 2011; Oliva *et al.*, 2014). However, while starvation is theoretically possible, it remains unclear how often it actually occurs (McDowell and Sevanto, 2010; Sala *et al.*, 2010, Hartmann, 2015).

Non-structural carbohydrates (NSCs) are frequently measured to test whether carbon starvation is occurring or was responsible for tree death (e.g. Bamber and Humphreys, 1965; Adams *et al.*, 2013; Hartmann *et al.*, 2013*a*). In trees, NSCs often make up the vast majority of the carbon storage pool (Hoch *et al.*, 2003) – carbon that can be remobilized for use in metabolism – and are used as indicators of a plant’s carbon balance (Körner, 2003, Hoch, 2015). During starvation, NSC levels decline (Hartmann *et al.*, 2013*b*; Sevanto *et al.*, 2014), but it is unclear at what NSC levels tissues die of starvation. Death from starvation may occur before NSC is completely depleted because sugars perform roles other than storage (McDowell and Sevanto, 2010; Galvez *et al.*, 2013; Hoch, 2015) or because substantial amounts of NSC may not be accessible (‘sequestered’ *sensu*Millard *et al.*, 2007). It is also unknown whether minimum NSC levels vary between organs or with environmental conditions. The ratio of living to dead cells, the amount of sugars fulfilling non-storage roles, or the amount of inaccessible reserves could all affect the amount of NSC that can be remobilized. As these factors could all vary with tissue type and potentially with growing conditions or plant size, NSC concentrations indicative of starvation could also vary. We therefore need to understand how variable minimum NSC levels can be (and why) in order to use tissue NSC concentrations as indicators of starvation.

Another aspect of starvation that is unknown is whether NSC depletion and starvation occur simultaneously and uniformly throughout the plant, killing the whole plant at once, or whether the remobilization and redistribution of reserves are somewhat compartmentalized (Wiley *et al.*, 2016), leading to localized depletion and tissue death. Sala *et al.* (2010) pointed out that trees could starve during drought before depleting whole-plant reserves because inhibited carbon transport could lead to localized reserve depletion (such as in the roots), which could kill the tree. Inhibited carbon transport under drought may arise due to insufficient turgor pressure in the phloem (Sala *et al.*, 2010; Sevanto *et al.*, 2014); however, even in the absence of water stress, the rates of carbon remobilization and transport may not be sufficient to meet the demands of some tissues, particularly if reserves are located at a distance (e.g. in another organ). If tissues do not starve at the same time, this could indicate that there are limiting constraints on the remobilization and distribution of stored NSC under ‘pure’ carbon stress. Such a result would also suggest that NSC reserves throughout a tree are not equally available for use by any part of the plant (Wiley *et al.*, 2016).

Because NSCs provide a supply of carbon when current photosynthesis cannot meet a plant’s needs, the amount of NSC storage– relative to demands (e.g. respiration) – should also be indicative of a tree’s ability to survive severe carbon stress prior to reaching a potential NSC minimum. It is unclear, though, whether the relative (i.e. tissue concentration) or absolute size (i.e. NSC mass or ‘pool size’) of the storage pool is a better general measure of storage and plant carbon balance, and therefore which is more indicative of survival potential (Canham *et al.*, 1999; Ryan, 2011; Hoch, 2015). Both measures have been found to correlate with survival under deep shade or following defoliation (Webb, 1981; Canham *et al.*, 1999, Gleason and Ares, 2004; Myers and Kitajima, 2007; Poorter and Kitajima, 2007), but because NSC concentration and pool size are often correlated, it is not clear which is a better predictor of survival under carbon limitation. This distinction is important because if a larger NSC pool *per se* can improve the chance of survival, then bigger trees could have higher survival potential than smaller trees under carbon stress, even if they have similar NSC concentrations. On the other hand, differences in NSC storage may be irrelevant for survival potential if differences in size cause large differences in respiration rates.

In this study, we exposed aspen seedlings to complete darkness at two temperatures to improve our ability to recognize death caused by carbon stress. Specifically, we sought to determine (1) at what NSC concentrations organs starve to death, and (2) whether tissues are all maintained until all reserves are depleted – suggesting equal access among tissues to NSC pools – or whether tissue survival is variable within the plant and decoupled from the whole-plant NSC pool. In addition, we grew seedlings under different shade levels before exposing them to darkness to manipulate initial NSC concentrations and pool size in our seedlings; by doing so, we aimed to test (3) whether initial NSC concentrations, initial NSC pool size, or respiration rates (i.e. carbon demand) are better predictors of survival under severe carbon stress. We hypothesized that seedlings would starve before NSC was depleted, that organs would vary in NSC concentrations at death, and that these minimum concentrations would depend on environmental conditions such as temperature. Finally, we predicted that higher NSC concentrations rather than larger NSC pool size or lower respiration rates would be associated with longer survival time.

## Materials and methods

### Plant material

In May 2014, aspen seedlings (*Populus tremuloides* Michx.) were grown from seed collected from an open-pollinated source in the boreal mixed-wood forest of central Alberta (AB), Canada. After germination, seedlings were initially kept in a growth chamber at 20 °C at the University of Alberta, well-watered, and fertilized with 1 g l^–1^ 10-52-10 NPK (Agrium Advanced Technologies, Calgary, AB) at 2 and 3 weeks after germination, and then with 2 g l^–1^ 15-30-15 NPK with micronutrients (Agrium Advanced Technologies, Calgary, AB) weekly thereafter. At 6 weeks after germination, seedlings were moved to a greenhouse, transplanted into 1.2-l pots filled with a mineral topsoil (silty-loam texture) collected from the Crop Diversification Center in Edmonton, AB, allowing for easy and complete extraction of root systems. Seedlings were acclimated for 1 week before being moved outdoors.

Prior to total darkness treatments, seedlings were grown under different levels of light availability to manipulate initial NSC concentration and pool size. On July 8, 351 seedlings were moved outside into shade shelters representing a light- (*n*=113), medium- (*n*=115), and dark-shade treatment (*n*=111) for 2 months. Each shelter consisted of a 3.0 × 1.85 × 1.50-m plywood frame covered with shade cloth (black polyethylene fabric; Shade Rite, Green-Tek Inc., Janesville, WI, USA) set inside a ‘growth box’. Light levels at mid-day averaged 43%, 7.6%, and 2.6% of full light for the light-, medium-, and dark-shade treatments, respectively. Seedlings remained well-watered and were fertilized every other week with 2g l^–1^ 15-30-15 NPK until early August when fertilization was ended to encourage budset. On September 7, after seedlings had set bud but still had green leaves, seedling height was measured. Subsets of seedlings were then harvested (*n*=8–9 per shade level) to characterize initial seedling NSC and size at the onset of the darkness treatments.

### Darkness treatments

To subject seedlings to severe carbon stress, seedlings were moved on September 9 into one of two growth chambers kept in 24-h darkness: one at 20 °C and one at 28 °C. Throughout the study, seedlings were well-watered and monitored for the timing of leaf death, first stem death (i.e. the first sign of any partial stem necrosis), and subsequent stem death. After a seedling’s leaves had died, they were removed to prevent mold.

For each initial shade (light, medium, and dark) × temperature in darkness (20 ° and 28 °C) combination, seedlings were pre-assigned to be harvested when they reached the following stages in the darkness treatment (determined from observations of a pilot study): (1) leaf area half dead (LHD); (2) leaves all dead (LAD); (3) stem half dead, i.e. outer phloem necrotic (i.e. turned black) (SHD); (4) stem all dead (SAD); (5) 3 or 5 d after SAD (28 and 20 °C treatment, respectively); (6) 6 or 10 d after SAD; and (7) 9 or 15 d after SAD. Seedlings at 28 °C were harvested earlier at 3, 6, and 9 d after SAD as opposed to 5, 10, and 15 d at 20 °C because we expected mortality to proceed more quickly at the warmer temperature. An eighth group of seedlings from each shade treatment in the 20 °C chamber was also harvested at the same time that seedlings in the 28 °C chamber reached SHD in order to provide a NSC comparison between temperature treatments at a similar length of time in darkness (as opposed to at a similar stage). To ensure equal size distribution across harvest stages, all trees from each shade×temperature in darkness treatment were ordered by height and divided into five groups based on size. Within each size group, one tree was randomly assigned to each of the seven or eight harvests, resulting in about five seedlings per harvest per shade×temperature in darkness treatment.

To determine if trees were capable of recovery at different stages of starvation, an additional five trees per shade×temperature in darkness treatment were moved into a lighted growth chamber (20 °C) at the SHD and SAD stages. Doing so allowed us to define death functionally [i.e. when seedlings were no longer able to recover (resprout), even though cells may still be living; Anderegg *et al.*, 2012]. For the 20 °C darkness treatment, additional trees were moved into the light on November 20 and January 20 when stems ranged from less than half dead to more than half dead.

### Plant harvests and respiration measurements

At harvest, leaves were removed (if applicable) and, for the initial harvest, leaf area was measured with a LI-3000 leaf area meter (LI-COR, Inc., Lincoln, NE, USA). Soil was carefully washed off the roots, which were then patted dry with paper towels and allowed to air dry for ~5 min. Root volume was determined using gravimetric volume displacement by submerging the root system in a beaker of water on a digital scale (1 g = 1 cm^3^ volume).

At harvest, we also measured root and stem respiration rates as these could be important determinants of survival time under carbon stress. Respiration was measured using a LI-6400XT with the 6400-22L opaque conifer chamber (LI-COR, Inc., Lincoln, NE, USA). CO_2_ was set at 400 ppm, and the chamber temperature was set to the treatment temperature (20 ° or 28 °C) except for the initial harvest (half measured at 20 ° and half at 28 °C). Whole root systems were patted dry, allowed to air dry for up to10 min, and then inserted into the chamber. For stems, the entire stem was placed in the chamber for short seedlings (most dark-shade and shortest medium-shade seedlings). For taller seedlings, the chamber was clamped down on a 7.5-cm segment of the lower stem, which was later removed and weighed separately. For roots and stems, respiration was measured for 3 min after it had plateaued. All tissues were oven-dried at 100 °C for 1 h, dried at 70 °C for 1 week, and then weighed. Respiration is presented on a dry-weight basis from initial harvests through to the SHD and SAD stages for stems and roots, respectively.

On a subset of roots, we also used a redox indicator to verify our assumption that coarse white roots – as opposed to those that had turned brown/black – were still alive. We submerged slices of white-root segments in solutions of 0.5% tetrazolium (K & K Laboratories, Inc, Plainview, NY, USA), which is used in seed viability tests and turns red in the presence of respiration. In most cases, white roots stained pink around the outer edge of the phloem or showed pockets of pink staining throughout the phloem.

### NSC sampling and analysis

NSC analysis was generally restricted to seedlings sampled at the initial harvest through to the SAD (stem all dead) stage, as seedlings were effectively dead by this point (see Results). Live leaf-tissue NSC was analysed at the initial harvest (pre-darkness treatment), while dead leaf-tissue NSC was analysed at the LHD and LAD harvest stages (i.e. leaf area half dead and leaf area all dead) and at the post-LAD stage from leaves removed in the growth chamber from a subset of seedlings. Stems and root tissue were analysed separately unless there was insufficient tissue present (<0.08 g); in these cases (i.e. for most dark-shade and some medium-shade trees), roots and stems were combined. For some dark-shade seedlings, there was still not enough tissue even after roots and stem were combined; in these cases, if possible, the two smallest seedlings from a given harvest stage (e.g. stem half dead) were combined to make one sample, reducing the sample size from 5 to 4.

In addition to the samples above, root NSC levels of light-shade seedlings were further explored at the SAD stage and beyond. A sub-sample of white-root tissue (presumed to be living) was processed separately from the remainder of the root system. This was done to ensure that we had a measure of root NSC that did not include partially decayed tissue, because as starvation progressed much of the root system began to die and decay (see Results).

Soluble sugar and starch content of all plant tissues were determined following the method of Chow and Landhäusser (2004). Dried samples were ground to 40 mesh with a Wiley Mill. Sugars were then extracted in 80% ethanol, and their content was determined spectrophotometrically via a phenol–sulfuric acid assay. After extraction, starch in the remaining pellet was digested with α-amylase and amyloglucosidase (Sigma-Aldrich, St. Louis, MO), and the resulting glucose content was determined spectrophotometrically using glucose oxidase/peroxidase-*o*-dianisidine solution (Chow and Landhäusser, 2004) and converted to starch equivalent. Sugar and starch concentrations were calculated as percent of sample dry weight. NSC concentrations are the sum of starch and soluble sugars. Where root and stem NSC were analysed separately, plant (i.e. stem and root) NSC concentrations were calculated as the dry-weight weighted average of stem and root NSC concentrations, making them comparable to NSC concentrations of small seedlings where roots and stems were analysed together. Finally, plant NSC pool size (i.e. NSC mass) was calculated as the sum of stem and root NSC concentration multiplied by stem and root mass, respectively.

### Data analysis

Initial shade treatment effects on seedling characteristics were analysed by univariate ANOVA in SPSS Statistics 23 (IBM Corp). When unequal variance was detected by visual examination of residuals, data were either ln-transformed or analysed by Welch’s ANOVA. Significant differences between shade levels were tested using either Tukey’s HSD test or Welch’s test with Games–Howell *post hoc* comparisons.

Respiration rates, plant NSC concentrations, root weight, and root volume/mass during the darkness treatments were analysed as three-way ANOVAs with initial shade treatment, temperature in darkness treatment, and stage at harvest (e.g. SHD) as main effects. Data were ln-transformed when unequal variance was detected. Significant main effects were further explored with Tukey’s HSD tests. Significant (*P*<0.05) and marginally significant (*P*<0.10) interactions were further explored with ‘slice tests’ (i.e. linear contrasts testing one main effect at each level of the other main effect).

Stem and root NSC concentrations during starvation were analysed as described above, but only with medium- and light-shade seedlings. In addition, because so many medium seedlings at SAD did not have enough tissue to analyse stem and roots separately, seedlings at 3 or 5 d after SAD were included with SAD medium-shade seedlings to increase sample size.

To determine the effects of temperature during darkness and initial shade treatments on survival time, we compared the number of days for plants to reach the following stages: (1) leaf area all dead (LAD); (2) first stem death; (3) stem half dead (SHD); and (4) stem all dead (SAD). Due to violations of normality and the presence of outliers, these data were analysed as permutation-based ANOVAs using the ‘aovp’ function of the lmPerm package in R (R Core Development Team, 2013) with shade, temperature, and shade×temperature as effects. Significant differences between shade treatments (across temperatures) were determined using Tukey’s HSD tests.

Finally, to better understand the underlying causes of different survival times, we tested if differences in survival time *within* initial shade treatments were related to NSC mass, concentration, or respiration rates. This could not be done directly as we did not measure survival times and early NSC and respiration rates on the same seedlings. Instead, we used different aspects of plant size as proxies for NSC concentration, NSC mass, and respiration rates during the early stages of starvation and then related those to survival time. To do this, we first calculated Pearson’s correlation coefficients between plant size variables [height, stem mass, root mass, root specific volume (i.e. root volume/mass), and specific stem length (SSL, stem height/mass)] and NSC concentrations, NSC mass, and respiration rates for seedlings harvested during leaf death (i.e. early stages of carbon stress). We then calculated correlation coefficients between these plant size variables and survival time (i.e. days until SAD) for seedlings harvested at SAD or beyond to determine whether variables correlated with initial NSC concentration, NSC mass, or respiration rates were better predictors of survival time. Analyses were performed in JMP 12 (SAS, Inc.) unless stated otherwise.

## Results

### Initial seedling characteristics prior to darkness treatment

The initial shade treatments applied before darkness treatments reduced growth and NSC pool size as expected (Supplementary Tables S1, S2 at *JXB* online) but, unexpectedly, did not affect NSC concentrations (Table S2). At the onset of darkness treatment, light-shade seedlings had 5–16 times the NSC mass (stem + root NSC) of medium- and dark-shade seedlings. The decreasing NSC pool size with increasing shade was due to a decrease in total biomass and not to a decrease in the root:stem mass ratio, which actually increased with decreasing shade level (Supplementary Table S1). In contrast to pool size, NSC concentrations of stems, roots, and the whole plant (stem+roots) were unaffected by shade, although there was substantial variation within treatments (Supplementary Table S2). Leaf NSC concentrations displayed only subtle variation with shade treatment; light-shade seedlings had the highest levels, but only marginally higher than medium-shade seedlings, which had the lowest (Supplementary Table S2).

### Tissue death and recovery during darkness treatment

**Fig. 1.**
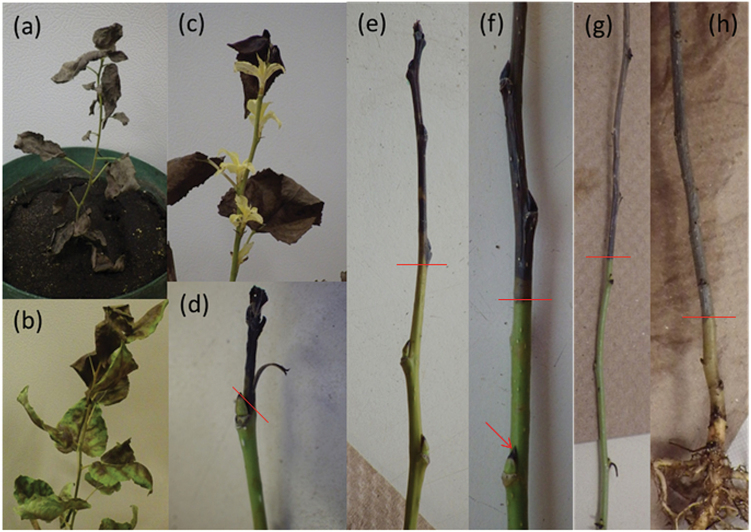
Above-ground tissue during starvation. (a) All leaf area is brown and dead. (b) All leaf area is dead, but some tissue is green but crisp. (c) Resprouting in the dark shortly after leaves have died. (d–h) Progression of stem dieback starting at the tip and proceeding downward to the base. Lines indicate the extent of necrosis. Note: green axillary buds are black-tipped (indicated by an arrow) and the root system in (h), after most of stem has died, appears to be still alive (i.e. not black).

**Fig. 2.**
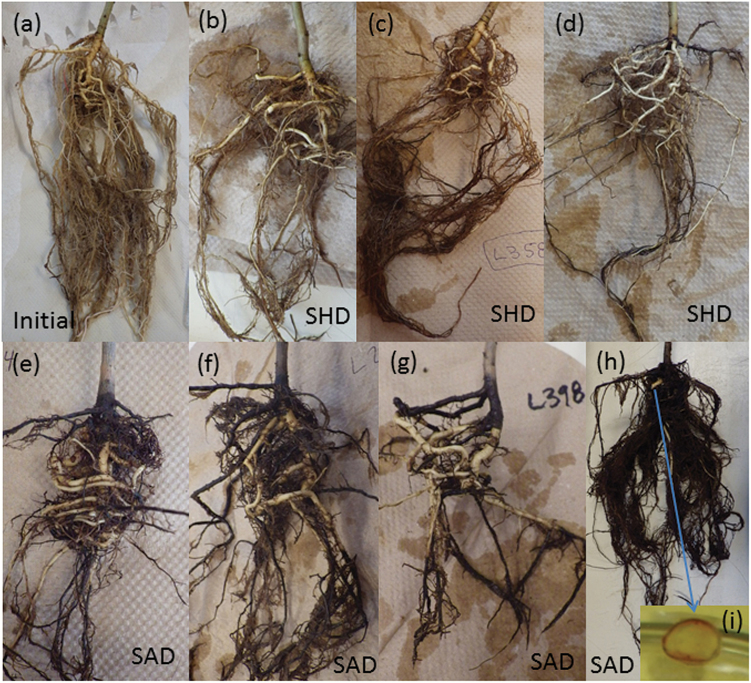
Roots of light-shade seedlings during starvation. (a) A healthy root system at the onset of starvation. (b–d) Root systems at the stem half dead stage (SHD). At this point there appears to be some fine-root death and loss and, in some cases, a small amount of coarse-root death. (e–h) Root systems at the stem all dead stage (SAD). The root condition is variable, but many have a substantial amount of dead major roots and dead or missing fine roots. (i) A cross-section of a white root from (h) was dyed by tetrazolium, thus indicating that this tissue was living.

Under darkness treatments, tissues did not die at the same time. Leaves generally died quickly (on average, after 9 and 18 d for 28 and 20 °C, respectively), before any stem death occurred (Fig. 1a). Stem death was gradual, starting at the terminal bud and preceding slowly to the base of the stem (Fig. 1d–h). Although harder to monitor, root systems also died back progressively. At later harvests, portions of the root system had turned black and were decomposing or had disintegrated, with finer roots appearing to die first (Fig 2). These observations were supported by significant declines in root mass and root specific volume between leaf death and when all the stem had died (SAD; Supplementary Figs S1, S2).

**Fig. 3.**
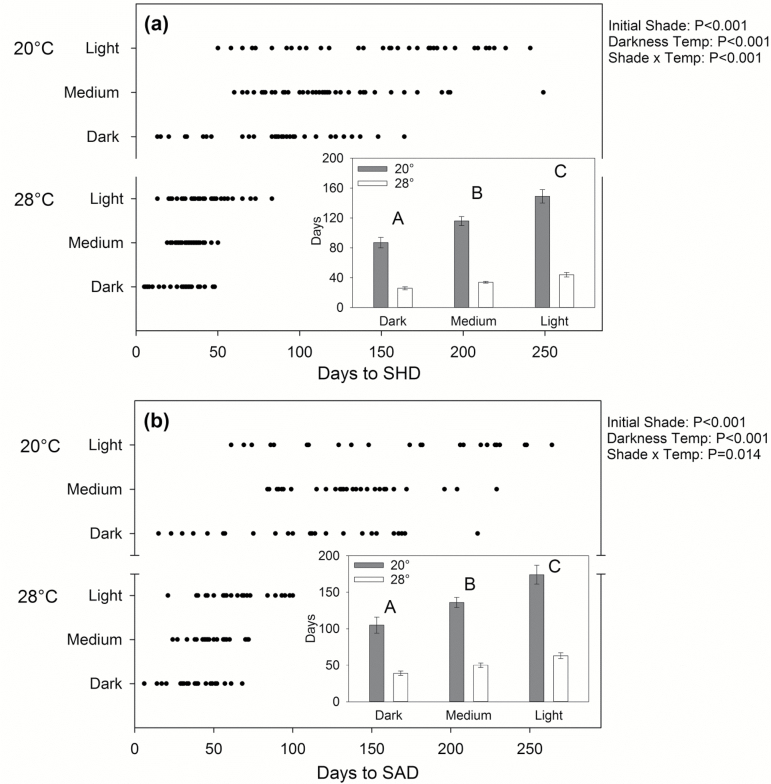
Darkness-temperature and initial (pre-darkness) shade treatment effects on survival time under darkness treatment. The number of days from the onset of darkness until (a) half the stem was dead (SHD) and (b) the whole stem was dead (SAD). Significant effects from permutation-based ANOVA are shown. Points represent individual seedling values. Inset graphs show treatment means ± SE, with different letters indicating significant differences between initial shade treatments across both darkness temperatures (i.e. main shade treatment effect) according to Tukey’s HSD. For sample sizes, see Supplementary Table S3.

Although tissue death proceeded similarly in all seedlings, temperature and initial shade treatments affected the time it took for seedlings to reach the different stages of dieback. Leaf death (LAD) occurred earlier at 28 than at 20 °C (9 versus 18 d; Supplementary Table S3), as did first stem death (16 versus 50 d; Table S3). The higher temperature also decreased the time until half (SHD) and all the stem (SAD) was dead by ~65–70% (Fig. 3, Supplementary Table S3). Time until SHD and SAD also decreased with increasing initial shade level, despite initially similar NSC concentrations (Fig. 3, Supplementary Table S3). Light-shade seedlings on average took 63 and 174 d to reach SAD at 28 and 20 °C, respectively, compared to only 39 and 105 d for dark-shade seedlings. Interestingly, there was large variation in survival times among seedlings within each shade treatment, leading to substantial overlap between shade treatments as well as between starvation stages (e.g. SHD versus SAD).

When seedlings were moved back into the light, recovery was possible through to the SHD stage (Supplementary Table S4). Survival was nearly 100% for seedlings with less than half the stem dead. At SHD, survival was still possible (27% survival at 28 °C, 40% at 20 °C across all initial shade treatments) but was particularly low for dark-shade seedlings, of which only 1 of 10 from both temperature treatments recovered. At SAD, no seedling recovered regardless of shade treatment; we therefore considered seedlings to be functionally dead at this point.

### NSC dynamics and respiration during darkness treatment prior to death

**Fig. 4.**
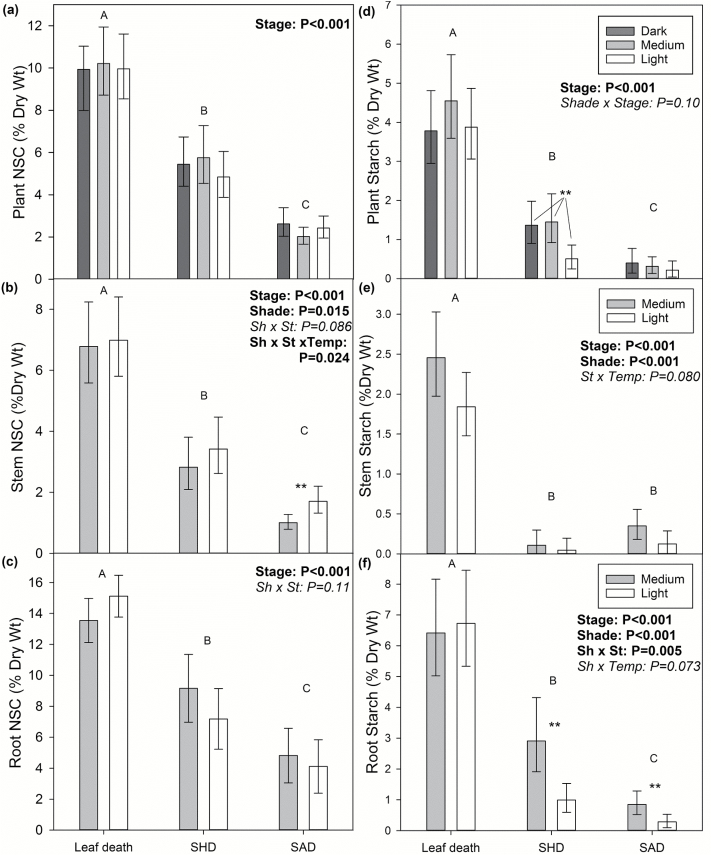
Plant, stem, and root non-structural carbohydrate (NSC) concentrations throughout starvation. (a) Total NSC and (d) starch concentrations for whole plants (i.e. roots + stem) for dark-, medium-, and light-shade treatments at the following stages of starvation: during leaf death (*n*=19, 20, 21), at stem half dead (SHD; *n*=11, 9, 10), and at stem all dead (SAD; *n*=8, 13, 11). (b, c) Total NSC and (e, f) starch concentrations for stems and roots separately for medium- and light-shade seedlings during leaf death (*n*=19,21), at SHD (*n*=8,10), and at SAD (*n*=13,13). Only significant ANOVA effects are listed. Different letters represent significant differences between stages of starvation across darkness-treatment temperatures and initial shade treatments. Asterisks indicate significant shade treatment effects at each stage (*P<0.05, **P<0.01, ***P<0.001). Values are marginal means calculated across both temperatures (all back-transformed except for root NSC), and error bars represent 95% CI. Temperature effects are not shown separately because main temperature effects were not significant.

During darkness treatments, plant (stem+roots) total NSC concentration declined, but neither temperature nor prior shade treatment had an effect on plant NSC levels at any given stage. Plant starch levels also declined during starvation; however, in contrast to total NSC, there was a significant shade effect at SHD, with light-shade seedlings having lower plant starch concentrations than dark- and medium-shade seedlings (0.5% versus 1.4%; stage×shade effect: *F*_4,104_=1.97, *P*=0.10; Fig. 4a).

Organ-specific NSC patterns indicate that a substantial amount of tissue death occurred before starch reserves were depleted. Stem NSC and starch concentrations declined over time, and when half the stem had died there was very little to no starch left above ground (at SHD, Fig. 4b). However, at this point root starch still averaged 1.3% for light-shade and 3.0% for medium-shade seedlings (Fig. 4c). Furthermore, at earlier stages of dieback, seedlings with stem dieback between 2.4–26% of stem length still had starch levels ranging from 0.4–1.6% in stems and 4.5–6.5% in roots (*n*=3 each for light- and medium-shade seedlings at 20 °C, harvested when 28 °C seedlings reached SHD; data not shown).

**Fig. 5.**
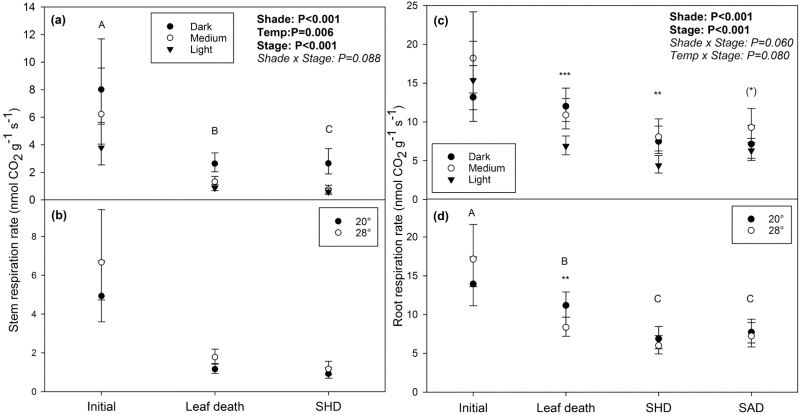
Stem and root mass-specific respiration rates throughout starvation by initial, pre-darkness shade treatment and temperature of darkness treatment. Mass-specific stem (a, b) and root (c, d) respiration were measured during harvests for initial measurements (i.e. pre-darkness), during leaf death, and when the top half of the stem had died (SHD). Root respiration was also measured when the whole stem had died (SAD). Means are back-transformed and error bars represent 95% CI. Only significant or marginal effects from the complete three-way ANOVA (stage, initial shade, temperature) are listed. D, M, and L indicate the initial dark-, medium-, and light-shade treatments, respectively. Different letters represent significant differences between stages of starvation across darkness temperatures and initial shade treatments. Asterisks indicate significant temperature (b, d) or initial shade treatment effects (a, c) at each stage: (*)*P*<0.10, *P<0.05, **P<0.01, ***P<0.001. For each shade treatment×temperature combination, *n*=4–5 at initial harvest, *n*=10–11 at leaf death, *n*=5–7 at SHD, and *n*=3–7 at SAD.

Stem and root respiration also declined over time during the darkness treatments (Fig. 5a, d; stage effects: *F*_2,95_=67.6, *P*<0.001 and *F*_3,127_=24.7, *P*<0.001 for stem and roots, respectively). The temperature of the darkness treatment, however, did not have a consistent effect on respiration, with only stem respiration showing a sustained increase under the higher temperature. Mass-specific stem respiration was 41% higher at 28 °C across all stages (Fig. 5b; temperature effect: *F*_1,95_=7.78, *P*<0.01). In contrast, mass-specific root respiration – although initially tending to be higher at 28 °C – was actually higher at 20 °C during leaf death, leading to a lack of a consistent temperature effect overall (Fig. 5d; temperature effect: *F*_1,127_=1.05, *P*=0.31).

Respiration during the darkness treatments also increased with increasing prior shade level. For stems, medium-shade seedlings respired at half and light-shade seedlings respired at one-third the rate of dark-shade seedlings across all stages (Fig. 5a; shade effect: *F*_2,95_=35.21, *P*<0.001). Light-shade seedlings also generally had significantly lower root respiration rates than dark- and medium-shade seedlings (Fig. 5c; shade effect: *F*_2,127_=11.70, *P*<0.001).

### NSC concentrations at tissue death

**Fig. 6.**
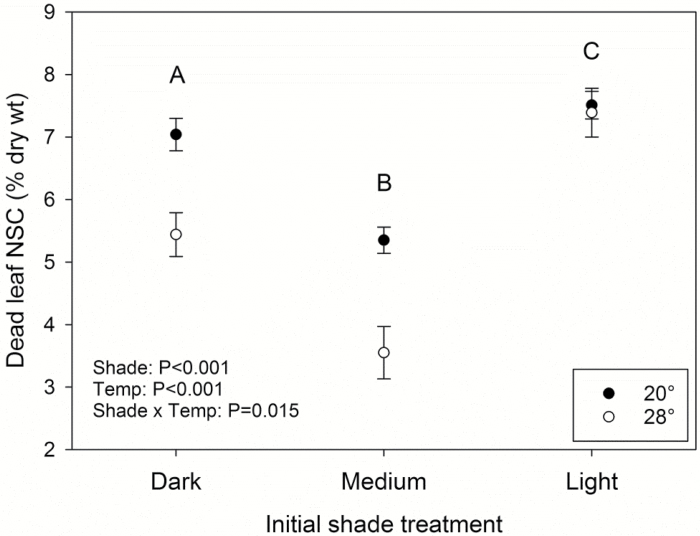
Non-structural carbohydrate (NSC) concentrations of dead leaves. Different letters represent significantly different values between the initial, pre-darkness shade treatments according to Tukey’s HSD, and error bars represent SE. Closed and open symbols represent seedlings from the 20 and 28 °C darkness treatments, respectively. Dead leaf tissue was collected at leaf area half dead, leaves all dead (LAD), and post-LAD (data were combined as there was no effect of stage on dead leaf NSC; *P*=0.55). *n*=14, 17, and 30 for dark-, medium-, and light-shade treatments, respectively.

Leaves died with variable, but surprisingly high sugar concentrations. Dead leaf NSC (all sugar) was significantly lower at 28 °C, although this was not consistent across all shade levels (Fig. 6). Overall, leaves from light-shade seedlings had significantly higher NSC at death than leaves from dark-shade seedlings, which had significantly higher concentrations than medium-shade seedlings; interestingly, this pattern mirrored the shade treatment differences in specific leaf area (i.e. light<dark<medium; Supplementary Table S1).

Stem NSC concentration at death was much lower than that of leaves, but like leaves it differed between shade treatments, with slightly higher NSC in the larger, light-shade seedlings (SAD stage in Fig 4b). The pattern of higher stem NSC at death in larger seedlings also occurred within the light-shade treatment: NSC concentrations of dead stems increased with increasing stem mass (*R*^2^=0.59, *P*=0.005). However, there was no relationship between stem mass and NSC concentration in medium-shade seedlings, (*P*>0.10). In light-shade seedlings, the upper and lower stem had remarkably similar NSC concentrations at death, and although there was a trend for lower NSC at 28 °C, the difference was not significant (Supplementary Fig. S3).

**Fig. 7.**
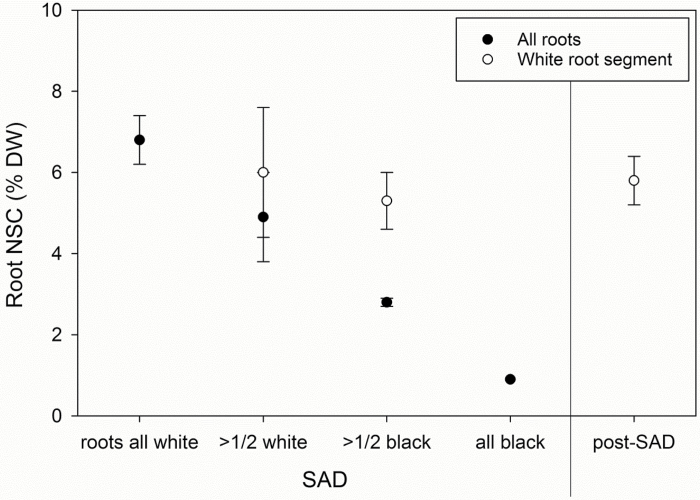
Non-structural carbohydrate (NSC) concentrations of whole-root systems versus segments of white roots for plants pre-treated with light-shade at stem all dead (SAD) and after SAD. Seedlings harvested at SAD (*n*=13) from both darkness temperature treatments were separated by the status of their coarser roots. White roots were assumed to be living, while black roots were dead and often partially decayed. Three seedlings with ‘roots all white’ had ~1 cm of live stem left. Three seedlings had >1/2 of coarse roots still white, and six seedlings had >1/2 black. One seedling had a completely black root system. Post-SAD white-root segments were collected from seedlings harvested 3–15 d after SAD occurred (*n*=10). White-root segment NSC did not significantly differ between SAD and post-SAD (*P*=0.72) or between root systems >1/2 white and >1/2 black at SAD (*P*=0.66). Error bars represent SE.

Root NSC concentrations at root death were difficult to determine because roots began to decompose and disintegrate in the soil medium. At and after the SAD stage, NSC concentrations of whole-root systems in light-shade seedlings declined with increasing degree of root death (Fig. 7), but these values are deceptively low as much of the root system had already partially decayed (Fig. 2). In contrast, subsamples of white segments taken from these same root systems indicated that ~5–6% NSC (0.3–0.4% starch) remained in the live roots when either little or most of the coarse roots were necrotic and decaying. However, in two cases, white-root segments contained between 1 and 2% starch.

### Role of NSC and respiration in survival time

There was substantial variation in survival time (time to SAD) within each initial shade treatment (Fig. 3), and the larger seedlings from each shade treatment generally survived longer under darkness, except in the medium-shade treatment (Table 1). For dark-shade seedlings, survival time was positively correlated with height and final stem mass and negatively correlated with specific stem length (SSL) and root specific volume (Table 1a). These size variables were in turn associated with both higher NSC concentration and pool size during the early stages of starvation (i.e. during leaf death), except for root specific volume, which was positively correlated with root respiration rate at 28 °C (Table 1a). Among light-shade seedlings, survival time was best predicted by stem mass (positive) and SSL (negative) at both temperatures. In addition, at 28 °C height and root mass were positively correlated and root specific volume was negatively correlated with survival time (Table 1b). Similar to dark-shade, most of the size variables positively related to survival time were also associated with initially higher NSC mass and concentration and lower root respiration, except for height, which was only correlated with NSC mass (Table 1b). For medium-shade seedlings, survival time was only correlated with root specific volume at 28 °C (negatively; Table 1c), and lower root specific volume was most strongly associated with initially higher NSC concentration and lower root respiration rates.

**Table 1.** Correlation coefficients between size variables and survival time for seedlings harvested at SAD (stem all dead) or beyond, and between size variables, plant non-structural carbohydrate (NSC), and mass-specific respiration rates for seedlings harvested during leaf death

	Survival time		Early starvation NSC and respiration			
	Days to SAD, 20 °C	Days to SAD, 28 °C	NSC mass (g)	NSC conc. (%)	Root respiration	Stem respiration
**(a) Dark-shade size traits**	(*n*=24)	(*n*=23)				
Height	0.45	0.65	0.71	*0.43*	NS	NS
Stem mass	0.59	0.74	0.91	0.58	NS	NS
SSL	–0.74	–0.58	–0.75	–0.54	NS	*0.44*
Root mass	NS	NS	0.90	0.54	NS	NS
Root specific volume	NS	–0.65	NS	NS	0.56	NS
**(b) Light-shade size traits**	(*n*=25)	(*n*=27)				
Height	NS	0.44	*0.41*	NS	NS	NS
Stem mass	0.59	0.60	0.85	0.53	–0.61	NS
SSL	–0.74	–0.55	–0.80	–0.74	0.86	NS
Root mass	NS	0.66	0.98	0.78	–0.66	NS
Root specific volume	NS	–0.67	–0.55	–0.58	0.55	*0.42*
**(c) Medium-shade size traits**	(*n*=27)	(*n*=27)				
Height	NS	NS	0.83	NS	NS	0.67
Stem mass	NS	NS	0.95	NS	NS	–0.69
SSL	NS	NS	–0.84	NS	NS	0.74
Root mass	NS	NS	0.96	NS	NS	–0.66
Root specific volume	NS	–0.47	*–0.38*	–0.69	0.53	NS

Correlation coefficients are only shown for *P*≤0.10, with values in *italics* indicating marginally significant correlations (0.05<*P*≤0.10). Height was measured at the onset of the darkness treatment. Stem and root mass, root specific volume (root volume/root mass), and SSL (specific stem length; stem height/stem mass) were measured (1) during leaf death for correlations with plant NSC and mass-specific respiration rates and (2) at SAD or beyond for correlations with survival time. *n*=19, 21, 20 for correlations with NSC and respiration for dark-, light-, and medium-shade respectively.

## Discussion

### Mortality under carbon stress

Under darkness, tissues died back gradually as opposed to all at once, indicating that the NSC storage pool in aspen seedlings cannot be represented as a single pool to which all tissues have equal access. As the stem underwent dieback from the top down, substantial starch reserves were still present in the roots (Fig 4c); this starch must have been available for remobilization (i.e. not sequestered) because root starch levels further declined to near zero by the time the whole stem had died. The fact that roots still contained >4% starch levels when the stem had already begun to die-back indicates that either a relative autonomy of reserves exists (i.e. no readily ‘shared’ reserves; tissues must rely on their own) or that the rate of NSC remobilization and transport to starving tissues is insufficient. It is thought that water stress may limit remobilization and phloem functioning, leading to local reserve depletion and death before whole-plant carbon reserves are depleted (Sala *et al.*, 2010; Hartmann *et al.*, 2013*a*; Sevanto *et al.*, 2014); our data suggest that limitations to NSC remobilization or transport may even occur in the absence of water stress.

The gradual progression of stem and root dieback during carbon stress is strikingly similar to the symptoms observed in decline-diseases where gradual canopy and root dieback occur as the result of interacting biotic and abiotic, inciting and contributing stressors (Manion, 1981; Houston, 1984). In these declines, initial dieback is sometimes viewed as an adaptive response to stress, such as drought (Houston, 1984; Rood *et al.*, 2000). Similarly, dieback during starvation might be an adaptive mechanism to limit respiratory losses, particularly in a species such as aspen, where sprouting and root suckering can be prolific. However, the earlier death of distal tissues (finer roots, leaves, tips of buds, and upper stem) may not be an active or even adaptive response, but may simply arise because these tissues have higher respiration rates and/or are the greatest distance from the main source of carbon storage (e.g. coarse roots in mature aspen; Landhäusser and Lieffers, 2012). In any case, if too much dieback occurs in response to starvation, trees may enter a spiral of decline (Manion, 1981), where the increasing loss of peripheral tissues, including the finer roots that provide water and nutrients necessary for photosynthesis, only exacerbates carbon stress (Landhäusser and Lieffers, 2012).

### NSC concentrations of dead tissues

Our experiments demonstrated that severe carbon stress leads to a significant but incomplete depletion of NSC, and starved tissues can retain a substantial level of carbohydrates at death. These lower limits of NSC can vary with organ and, to a certain extent, with current (e.g. temperature) and previous (e.g. shade) growth conditions. This variability suggests that there may not be one universal concentration indicative of starvation, which complicates the inference of starvation from examination of NSC concentrations. The variation in NSC was almost all due to sugars, the concentration of which could be well above zero. Although sugars have been reported to approach near zero under low CO_2_ in Norway spruce seedlings (Hartmann *et al.*, 2013*b*) and in glucose-starved tissue cultures (Brouquisse *et al.*, 1991), other studies have reported levels of 1–2% for light-deprived pinyon pine and Douglas fir (Marshall, 1984; Sevanto *et al.*, 2014), which is consistent with our stem sugar concentrations, but much lower than our leaf sugar levels. Root NSC levels were difficult to assess because of the continuous and increasing degree of decomposition of dead roots, but the 5–6% sugar measured in undecayed (white) roots surrounded by dead and decaying root systems suggests that the minimum NSC concentration for roots may be in this vicinity. Care must be taken when sampling roots for NSC measurements during the mortality process and under stress, since a large amount of NSC might be lost quickly through decomposition as root systems die gradually, particularly when growing in soil.

Relatively high NSC levels at death are often treated as evidence against starvation or carbon limitation, but our data show that relatively high NSC levels can occur in starved plants. For leaves, NSC concentrations at death measured in our experiment (shade treatment averages: 4–7.5%) are well within the measured range of values of dead leaves from droughted pinyon pine (4%; Adams *et al.*, 2013) and lodgepole pine attacked by mountain pine beetle (8.4%; Wiley *et al.*, 2016), suggesting that such relatively high NSC concentrations cannot rule out starvation as a potential mortality mechanism. Our starved leaf NSC concentrations are also similar to values reported in abscised leaves of birch and oak seedlings in autumn (Palacio *et al.*, 2013), although lower than the 14% reported for mature, abscised aspen leaves (Landhäusser and Lieffers, 2003). However, comparisons of absolute NSC values should always be made with caution, as measurements between laboratories can be so variable (Quentin *et al.*, 2015).

Complete sugar depletion during starvation may not occur for a variety of reasons, and further work will be necessary to determine why the degree of depletion varies. A certain amount of sugars may be unavailable to metabolism because they are functioning in other, non-storage roles such as osmotic adjustment and hydraulic maintenance, and this amount may differ between organs and could change with plant size or growing conditions such as degree of water stress (Sala *et al.*, 2012; Dietze *et al.*, 2014; Sevanto *et al.*, 2014). Leaves, for example, generally have lower water potentials than other organs, and their cells may require more sugar to function as osmotica than stems. Alternatively, it is possible that all *cells* have a similar minimum NSC concentration, but *tissue* minima differ because some tissues, such as leaves, have a greater proportion of living cell types than others, such as woody tissues. Finally, if remaining carbon stores are not located in the cells that are starving, as discussed above, insufficient remobilization or transport rates could lead to the death of a critical proportion of cells before all potential reserves have been used up within a tissue. Therefore, differences in NSC concentration at death may result from differences in carbon remobilization or transport rates that could vary with plant size, organ type, or environmental conditions. For example, we found that in some cases, higher temperature reduced NSC concentrations at death (Fig. 6). Higher temperatures could theoretically increase remobilization and/or transport rates by increasing enzymatic activity, increasing diffusion rates of sugars from storage cells into starving cells, or by increasing phloem sap velocity via decreased sap viscosity. Similarly, the trend of higher minimum NSC concentrations in bigger seedlings and thicker tissues (e.g. increasing stem NSC with stem mass and increasing leaf NSC with lower SLA) could result from potentially slower sugar transport rates between some storage sites (e.g. xylem parenchyma or pith) and major metabolic sites (e.g. cambium) as diffusion rates decrease with increasing distance.

In contrast to sugars, starch was generally depleted by the time tissues died and may be a more reliable hallmark of starvation. Starch depletion in starved tissues has been reported in conifers under dark treatment or CO_2_ deprivation (Marshall, 1984; Hartmann *et al.*, 2013*b*) as well as in starved tissue cultures (Brouquisse *et al.*, 1991), although Piper and Fajardo (2016) reported about 1% starch remaining in darkened *Acer* seedling tissues at death. Of course, starch depletion may not always be possible, and in our experiment there were instances when starch was still present in dead tissues, although this was uncommon. Some researchers have suggested that large starch deposits may become inaccessible in trees (Millard *et al.*, 2007), particularly under water stress (Sala *et al.*, 2012; Sevanto *et al.*, 2014). However, under these conditions, death may not occur via starvation, and currently it is not clear whether sequestration is at all common, as even carbon reserves more than a decade old may be remobilized following severe disturbance (Carbone *et al.*, 2013).

### The role of respiration and NSC storage in survival time under starvation

Longer survival times were expected in the darkness treatment at 20 °C due to lower respiration rates, which were observed for leaf (data not shown) and stem tissue but not roots. The lack of higher root respiration at 28 °C may result from acclimation. At high soil temperatures, after an initial increase, root respiration can decline to levels lower than those prior to high-temperature exposure (Huang *et al.*, 2005, Di Iorio *et al.*, 2016). However, our harvested root systems often contained dead roots, and this could obscure or artificially create differences in root respiration rates between temperatures if treatments systematically differed in the amount of attached, dead roots. Inclusion of dead roots in respiration measurements could (1) reduce apparent mass-specific respiration rates because total root mass includes non-respiring roots, or (2) increase apparent mass-specific respiration rates if dead roots were decomposing and releasing CO_2_ at a higher rate than root respiration.

Differences in survival time between initial shade treatments were also related to differences in respiration, in contrast to our prediction that survival time would be driven by differences in initial NSC concentrations. Differences in respiration may also have been partly responsible for variation in survival time *within* light- and medium-shade treatments too, as size variables that were positively correlated with survival time were associated with lower root respiration rates (Table 1b, c). Because reducing carbon loss through respiration is a well-known strategy for coping with low-light conditions (Grime, 1965; Loach, 1967), we would have expected dark- and medium-shade seedlings – which grew under deeper shade – to have the lowest (or at least similar) respiration rates. Instead, the lower respiration rates of light-shade seedlings allowed them to live longer off the same initial NSC concentration as the medium- and dark-shade seedlings. However, the lower respiration rates of the larger, light-shade seedlings may be explained by their size, rather than as an expression of tissue acclimation. The lower specific stem length (SSL) of light-shade seedlings (Supplementary Table S1) indicates that they had thicker stems, and SSL was positively correlated with mass-specific stem respiration across shade treatments during leaf death (*R*^2^=0.53, *n*=54, *P*<0.001). Mass-specific respiration rates commonly decline with increasing diameter in roots, stems, and branches (Desrochers *et al.*, 2002; Cernusak *et al.*, 2006; Makita *et al.*, 2009, 2012; Sendall *et al.*, 2015), and this decline is associated with a decrease in the ratio of living cells or meristematic tissue to dead xylem with increasing diameter (Pregitzer *et al.*, 1998; Damesin *et al.*, 2002).

Because we were unable to manipulate initial NSC concentration as expected with the pre-darkness shading treatments, it remains unclear whether initially greater NSC mass – independent of concentration – increased survival time. Although we found a pattern of increasing survival time with increasing initial NSC mass between initial shade treatments, the longer survival time may have been caused by the decreasing respiration rates with increasing shade level (which means that NSC concentration per unit respiration also declined with increasing shade level). In addition, because NSC mass and concentration were generally correlated *within* dark- and light-shade treatments, both NSC measures were correlated with the size variables that predicted survival time (Table 1a, b), making it impossible to distinguish between potential effects of mass versus concentration in these treatments. This perhaps speaks to the highly correlated nature of NSC mass and concentration, at least in seedlings. However, an argument against greater NSC mass as a cause of prolonged survival is that in the medium-shade treatment, where height, stem and root mass and SSL were *only* correlated with NSC mass, these size variables were not significant predictors of survival time (Table 1c); but they should have been if NSC mass was an important determinant of survival time. Instead, the only trait related to survival time was specific root volume, which was the only size trait correlated with NSC concentration and root respiration (Table 1c).

## Conclusions

Mortality induced by severe carbon stress (i.e. starvation in darkness) in aspen seedlings occurs as a gradual process of dieback – strikingly similar to decline-diseases initiated by drought and/or defoliation. This process commences long before whole-plant reserves are depleted, suggesting that reserve mobilization and transport may be important factors limiting survival under carbon stress. At death, tissues generally contained no starch, but substantial levels of sugars were still present. Further, sugar concentrations at death varied with organ type and previous (i.e. shade treatment, perhaps reflecting differences in plant size) and current environmental conditions (i.e. temperature). Such variability complicates efforts to identify critical NSC thresholds below which trees starve; however, understanding the variation in minimum NSC levels – and its causes – will be necessary if we continue to use NSC concentrations as indicators of carbon limitation during stress and after mortality in trees.

## Supplementary data

Supplementary data are available at *JXB* online.

Table S1. Shading effects on initial size and biomass.

Table S2. Shading effects on initial NSC.

Table S3. Temperature/shade effects on the timing of mortality stages.

Table S4. Seedling recovery following darkness.

Fig. S1. Root mass during starvation.

Fig. S2. Root specific volume during starvation.

Fig. S3. Upper versus lower stem NSC concentrations.

## Supplementary Material

Supplementary Tables_S1_S4_Figures S1_S3
