# Supplementary material for: Dying piece by piece: carbohydrate dynamics in aspen (Populus tremuloides) seedlings under severe carbon stress
Source: J Exp Bot. 2017 Sep 27;68(18):5221–32. doi: 10.1093/jxb/erx342 (PMC5853906; doi:10.1093/jxb/erx342)
Supplement: Supplementary Tables_S1_S4_Figures S1_S3 [file erx342_suppl_supplementary_tables_s1_s4_figures_s1_s3.pdf]

**Supporting Information for**

# Dying piece by piece: carbohydrate dynamics in aspen seedlings under severe carbon stress and starvation

Erin Wiley, Günter Hoch & Simon M. Landhäusser

**Table S1: Effects of initial shading on seedling size and biomass allocation prior to darkness treatment**

| | Initial Shade Level | | | Shade Effect | |
|---|---|---|---|---|---|
| | *Light* | *Medium* | *Dark* | *F* | *P* |
| Height[1,2] (cm) | 53.6 [51.0-56.4][a] | 24.8 [23.4-26.3][b] | 10.9 [10.2-11.6][c] | 26.01 | <0.001 |
| Total mass[1] (g) | 4.79 [2.59-8.85][a] | 0.99 [0.57-1.71][b] | 0.33 [0.26-0.43][c] | 43.62 | <0.001 |
| Leaf area[1] ($cm^2$) | 539 [288-1012][a] | 200 [125-324][b] | 62 [42-94][c] | 25.75 | <0.001 |
| Root: stem[1] (g/g) | 0.78 [0.61-1.01][a] | 1.12 [0.87-1.44][ab] | 1.60 [1.26-2.03][b] | 9.16 | 0.001 |
| SSL[3] (cm/g) | 35±6[a] | 123±16[b] | 212±9[c] | 70.7 | <0.001 |
| SLA[4] ($m^2$/g) | 274±34 [a] | 366±47 [b] | 335±74 [ab] | 10.21 | 0.002 |

[1]Averages and 95% CI are back-transformed

[2]Height averages are based on all live trees (N>100 for each treatment), all other averages are based on trees harvested pre-darkness (dark: N=9; medium: N=8; light: N=8)

[3]SSL=specific stem length (i.e. stem height/mass)

[4]SLA= specific leaf area

**Table S2: Effects of initial shading on seedling nonstructural carbohydrates**

| | **Initial Shade Level** | | | **Shade Effect** | |
|---|---|---|---|---|---|
| | *Light* | *Medium* | *Dark* | *F* | *P* |
| Plant NSC mass[1] (mg) | 338 [189-604][a] | 57 [32-102][b] | 15 [8-25][c] | 33.1 | <0.001 |
| Plant %NSC | 12.5±0.7[a] | 13.8±1.6[a] | 11.6±1.4[a] | 0.55[†] | 0.59 |
| Plant % Sugar | 6.7±0.4[a*] | 6.4±0.4[a] | 5.5±0.3[a*] | 2.88 | 0.077 |
| Plant % Starch | 5.8±0.4[a] | 7.4±1.2[a] | 6.1±1.2[a] | 0.76[†] | 0.49 |
| Root %NSC | 16.2±0.9[a] | 16.1±1.8[a] | NA | 0.01[†] | 0.94 |
| Stem %NSC | 9.5±0.6[a] | 9.4±0.9[a] | NA | 0.01 | 0.99 |
| Leaf %NSC | 10.4±0.4[a*] | 9.2±0.3[a*] | 9.8±0.4[a] | 2.46 | 0.11 |

[1]Averages and 95% CI are back-transformed

†Welch's ANOVA

Different letters indicate significantly different pairwise comparisons (P<0.05), and paired asterisks indicate marginally significant differences (0.05<P<0.10).

Plant NSC mass and %NSC were calculated for roots and stem combined.

N=8,8, and 9 for light, medium and light shade, respectively

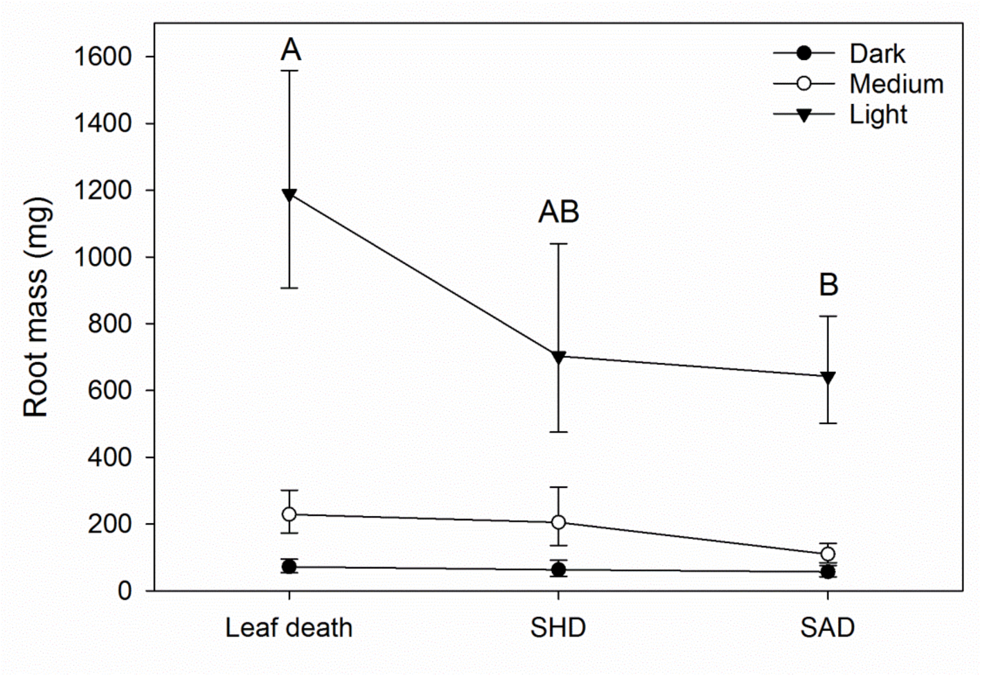


**Figure S1**. Decline in root mass during starvation from leaf death to stem half dead (SHD) to stem all dead (SAD). Averages are back-transformed (ln) and error bars represent 95% confidence intervals. Different letters represent statistically significant differences between harvest stages, across all initial shade treatments, according to Tukey's HSD. 3-way ANOVA effects: Stage: $P<0.0001$; Shade: $P<0.0001$; Temp & all interactions: $P>0.20$. N=20, 20, 21 at leaf death, N=11, 9, 10 at SHD, and N=8, 13, 13 at SAD for dark, medium, and light shade treatments, respectively.

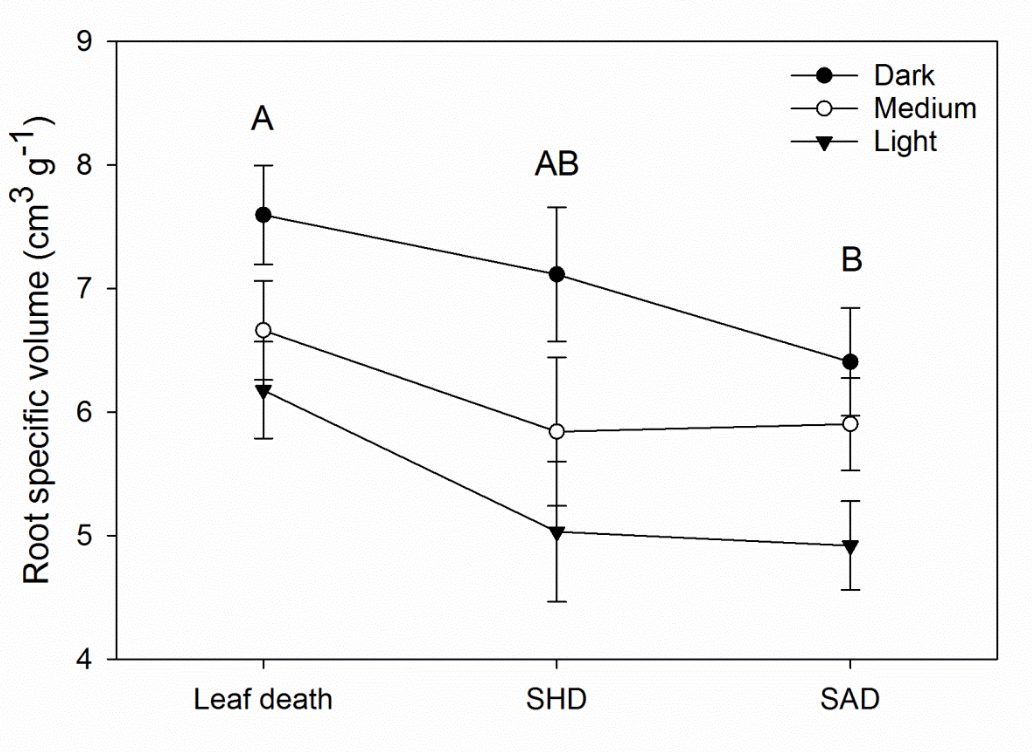


**Figure S2.** Decline in root specific volume during starvation from leaf death to stem half dead (SHD) to stem all dead (SAD). Error bars represent 1 SE, and different letters represent statistically significant differences between harvest stages, across all initial shade treatments, according to Tukey's HSD. 3-way ANOVA effects: Stage: P=0.004; Shade: P=0.040; Temp × Stage: P=0.001; Temp × Shade: P=0.017; Temp, Stage × Shade, Temp × Stage × Shade: P>0.14. The interaction between stage and darkness-temperature was explained by a significantly lower root mass/volume at 20° than at 28° at SHD and SAD stages, but not during leaf death. For sample sizes, see Figure S1.

**Table S3:** Darkness temperature and initial shade effects on the timing (i.e. days until) of mortality stages

| | Light | | | Medium | | | Dark | | | Temp | Shade | T × Sh |
|---|---|---|---|---|---|---|---|---|---|---|---|---|
| | **28°** | **20°** | | **28°** | **20°** | | **28°** | **20°** | | *P* | *P* | *P* |
| **LAD** | 9.0±0.2 [39] | 17.9±0.5 [53] | a | 7.9±0.3[42] | 17.1±0.6 [53] | a | 8.0±0.3 [37] | 18.5±1.1[41] | a | <0.0001 | 0.22 | 0.37 |
| **1st sd** | 13±1[39] | 45±5 [44] | a | 16±1[37] | 50±4 [48] | a | 18±1 [14] | 55±4 [20] | a | <0.0001 | 0.13 | 0.84 |
| **SHD** | 44±3 [36] | 149±9 [37] | a | 34±1 [41] | 116±6 [37] | b | 26±2 [34] | 87±7 [35] | c | <0.0001 | <0.0001 | 0.0004 |
| **SAD** | 63±4 [26] | 174±13[27] | a | 50±3 [28] | 136±7 [28] | b | 39±3 [26] | 105±11 [23] | c | <0.0001 | <0.0001 | 0.0142 |

LAD=Leaves all dead, 1st sd= first stem death, SHD= stem half dead, SAD=stem all dead. Values are averages ± SE. Effects of temperature and shade were analyzed using permutation-based ANOVA. Different letters within a row indicate significant differences between shade treatments across both temperatures (i.e. main effect differences) according to Tukey's HSD. Sample sizes (N) for each treatment combination are shown in brackets.

**Table S4.** Proportion of seedlings that recovered after return to the light measured on a subset of seedlings that were moved from the darkness back to the light when stems were half dead (SHD) and when stems were all dead (SAD).

| | **Recovery at < SHD**[1] | **Recovery at SHD** | | **Recovery at SAD** | |
|---|---|---|---|---|---|
| **Shade** | *20° C* | *20° C* | *28° C* | *20° C* | *28° C* |
| *Light* | 5/6* | 3/5 | 1/5 | 0/5 | 0/5 |
| *Medium* | 4/4 | 3/7* | 3/5 | 0/5 | 0/5 |
| *Dark* | 2/2 | 1/5 | 0/5* | 0/4 | 0/5 |
| *Total* | 11/12 | 7/17 | 4/15 | 0/14 | 0/15 |

[1]Additional trees at 20° C were moved when stems were less than half dead (<SHD) at either 72 or 132 days in darkness to ensure that seedlings were still alive. Recovery was assumed if seedlings resprouted or suckered from the roots, produced green leaves, and were able to continue growth.

* One seedling responded aboveground to light by beginning to flush or a change in pigmentation but then died (not counted as recovered).

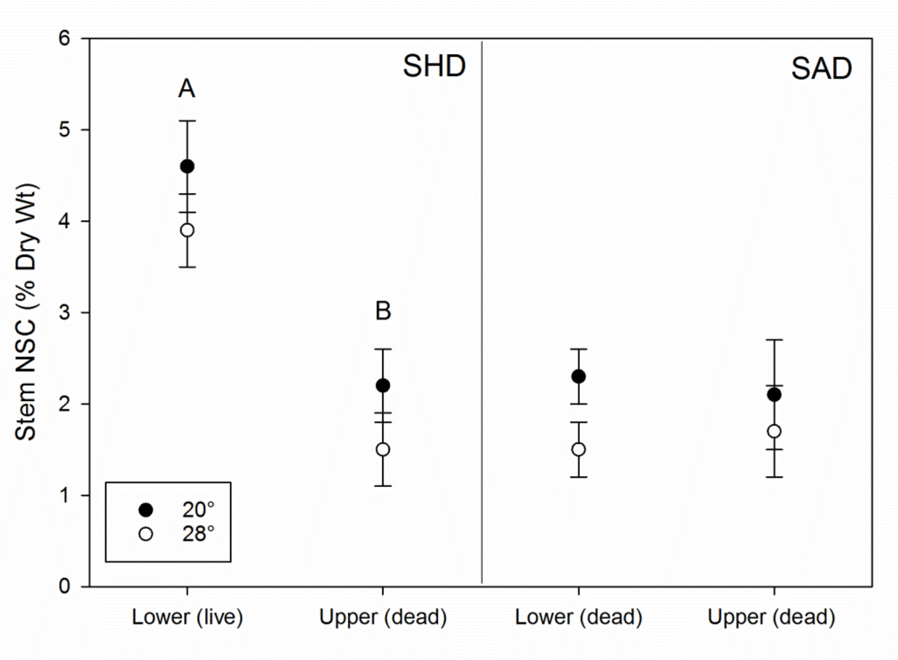


**Figure S3:** Upper versus lower stem NSC levels of light shade seedlings at SHD and SAD. Different letters represent significant differences between upper and lower stem NSC according to paired t-tests. Error bars represent standard error. Though there is a trend for higher NSC at 20° vs 28°, this trend was not significant (either by t-tests or multivariate ANOVA including upper and lower stem). N=5,5 (SHD) and N= 6,4 (SAD) for 20° and 28°, respectively.
